# Multicenter Assessment of Combined Brain and Cervical Spinal cord 3T MP2RAGE T1 Measurements for Reliable Tissue Microstructure Quantification

**DOI:** 10.1002/nbm.70350

**Published:** 2026-07-08

**Authors:** Malo Gaubert, Benoit Combès, Henitsoa Rasoanandrianina, Jean‐Christophe Ferré, Alice Dufey, Raphaël Chouteau, Bertrand Audoin, Jean Pelletier, Sarah Demortière, Anne Kerbrat, Elise Bannier, Virginie Callot

**Affiliations:** ^1^ Univ Rennes, CHU Rennes, (Service de radiologie et imagerie médicale) Rennes France; ^2^ University Rennes, Inria, CNRS, Inserm, IRISA UMR 6074, Empenn U1228 Rennes France; ^3^ Aix‐Marseille Univ CNRS, CRMBM Marseille France; ^4^ APHM, Hôpital Universitaire Timone CEMEREM Marseille France; ^5^ Univ Rennes, CHU Rennes, (Service de neurologie) Rennes France; ^6^ Aix Marseille Univ, APHM, Hôpital de la Timone Department of Neurology Marseille France

**Keywords:** brain, MP2RAGE, multicentre, repeatability, reproducibility, spinal cord, T1

## Abstract

Recent developments in quantitative T1 have enabled simultaneous scanning of both the brain and the cervical spinal cord (cSC) using the magnetization‐prepared two rapid acquisition gradient echoes (MP2RAGE) sequence. However, the multicentre variability of such an approach has not yet been assessed. In this paper, we aim to evaluate multicentre variability (inter‐scanner, between‐session, between‐participant) of T1 MP2RAGE measurements in the brain and cSC. A total of 52 MP2RAGE scans were acquired at two centres equipped with Siemens 3T MRI systems using an optimized MP2RAGE sequence. A subset of participants underwent scanning at both centres to evaluate inter‐site variability. Between‐session and inter‐participant variability were assessed both globally and within specific regions, including brain white matter, cortical gray matter, deep gray matter, and cervical spinal cord vertebral levels using a linear mixed effect model. Mean intra‐site/inter‐site/between‐participant coefficients of variation (COV) were 0.4/0.9/2.3% for the brain ROIs and 2.0/2.4/1.7% for the cSC. Our findings validate the consistency and reliability of the MP2RAGE brain and cervical spinal cord simultaneous acquisition in a multicentre setting. This approach has the potential to significantly impact the study of central nervous system pathologies, such as multiple sclerosis, by enabling fast, accurate, and reproducible quantitative imaging collection across multiple centres.

## Graphical abstract

Reliability of simultaneous brain and spinal cord magnetization‐prepared two rapid acquisition gradient echoes (MP2RAGE) T1 measurements was evaluated in a multicentre context. Mean intra‐site/inter‐site/between‐participant coefficients of variation (COV) were 0.4/0.9/2.3% for the brain ROIs and 2.0/2.4/1.7% for the cSC, confirming the potential of T1 MP2RAGE measurement for further multicentric applications focusing on CNS pathologies.

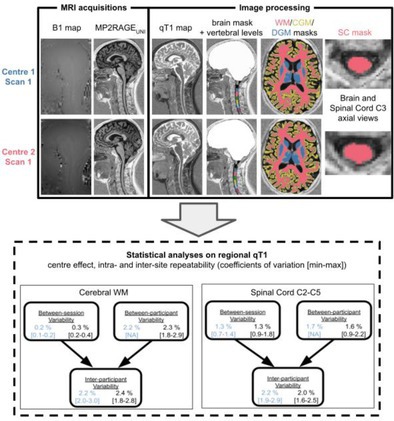

**Abbreviations**
CAT: computational anatomy toolbox
cGM: cortical gray matter
CNS: central nervous system
COV: coefficients of variation
cSC: cervical spinal cord
cSC C2–C5: C2 to C5 levels of the cervical spinal cord
dGM: deep gray matter
FoV: field of view
FSL: FMRIB Software Library
GRAPPA: generalized autocalibrating partial parallel acquisition
GRE: gradient echo sequence
MP2RAGE: magnetization‐prepared two rapid acquisition gradient echoes
MRI: magnetic resonance imaging
qT1: quantitative MP2RAGE T1
ROI: region‐of‐interest
SCT: spinal cord toolbox
SPM: statistical parametric mapping
TI: inversion time
TR: repetition time
WM: white matter

## 1 Introduction

The magnetization‐prepared two rapid acquisition gradient echoes (MP2RAGE) sequence introduced by Marques et al. was proposed in 2010 to provide T1‐weighted images, from which T1 maps can be derived using Bloch simulations [1]. It implements an adiabatic inversion pulse followed by two magnetization prepared gradient echo blocks with different inversion times (TI1, TI2) and low flip angles (ɑ1, ɑ2) generating two different contrast images (INV1, INV2). These two images can be combined in various ways to provide, among others, the MP2RAGE_UNI_ and quantitative T1 (qT1) contrasts, with reduced influence from proton density, T2* effect and reception field inhomogeneity. In comparison with other T1 relaxometry approaches such as the gold standard inversion recovery, saturation recovery, look locker or variable flip angle techniques, the MP2RAGE sequence enables robust isotropic millimetric T1 quantification in less than 10 min [1]. Initially proposed for brain imaging, recent optimisations allow for both brain and cervical spinal cord acquisitions simultaneously with a good trade‐off between acquisition time, robustness and accuracy [2, 3]. This sequence is of particular interest to investigate tissue microstructural modifications occurring in pathologies such as multiple sclerosis (MS) [4, 5] or degenerative cervical myelopathy and ageing [6, 7, 8]. Indeed, changes in T1 values can reflect several biological modifications, among which demyelination and inflammation processes [9]. However, the robustness of the technique in a multicentre setting must be established before its broader deployment. To this end, we conducted a reproducibility and travelling brain and spine study to evaluate the variability of the T1 measurements between sessions, across sites and between participants.

## 2 Material and Methods

### 2.1 Participants and Study Design

Data included in this work was collected in the context of the multicentric MSTRACTS (NCT04220814) and OSV‐IRM (NCT05107232) studies, approved by an Institutional Review Board. Thirty‐seven healthy control subjects from two centres were enrolled between 2021 and 2023. Mean [SD; range] age was 39.6 [11.2; 24–62] years old and the proportion of males/females was 10/27 (37% of males). Written informed consents were collected prior to data acquisition. The main inclusion criteria were the absence of any history of central nervous system (CNS) diseases or spinal cord trauma.

Of the 37 volunteers, 31 were seen once in a given centre only and six were seen at least once in each of the two centres. The exact repartition is presented in Table 1. Subjects who underwent a scan‐rescan in any centre were taken out of the scanner between each scan and the scanner adjustment parameters were reset.

**TABLE 1:** Summary of subject counts for each centre. The number of subjects scanned once (in one centre only) is displayed in blue squares and those with scan‐rescan (in the same centre or in both centres) are displayed in green squares. Fifty‐two scans were acquired in total.

Number of healthy controls		Scanned in Centre 2		
		0	1	2
**Scanned in centre 1**	**0**		8	1
	**1**	22	1	
	**2**		2	3

Scanned at least two times (in same centre or both centres) [*n* = 7]
Scanned in one centre only [*n* = 30]

### 2.2 Image Acquisition

Both recruiting centres were equipped with a 3T MRI scanner (Prisma with VE11 software in Centre 1, Vida with XA50 software in Centre 2) and a 64‐channel head/neck coil from the same manufacturer (Magnetom Siemens Healthineers, Erlangen, Germany). The same MR product sequences and parameters were used across the scanners. The protocol included a sagittal 3D MP2RAGE sequence [TR/TE 4000/2.48 ms, FoV 243 × 300 mm^2^, 176 slabs, 6/8 partial Fourier, 0.9 × 0.9 × 1 mm^3^ voxel size, TI1/TI2 = 650/2000 ms, ɑ1/ɑ2 = 4/5°, GRAPPA 2] and a sagittal 2D magnetization‐prepared turbo FLASH B1 + map [TR 15260, FoV 243 × 300 mm^2^, 35 slabs, 4.7 × 4.7 × 5.0 mm^3^ voxel size, ɑ = 8°], both covering the brain and cervical SC (cSC) [2]. 3D MP2AGE had 176 partitions and the 2D FLASH had 35 slices. The scan volumes were manually positioned to cover both brain and cervical spinal cord, with the centre of the FOV roughly at the same location (between the pons and the medulla) for each subject, the sagittal plane being aligned as much as possible with the cord in the coronal plane and the centre of the FOV at the magnet isocentre.

The composite volume MP2RAGE_UNI_, used to derive quantitative T1 map, was automatically generated online by the scanner based on the two GRE volumes acquired with the two different inversion times [1]. The B1 + map (flip angle map), used to correct a posteriori the T1 map from B1 + inhomogeneities, was also automatically generated by the scanner.

### 2.3 Image Processing

Image processing was performed using the Computational Anatomy Toolbox CAT12.8.2 (release 2170) [10], a toolbox of Statistical Parametric Mapping SPM12 (version 7771) used on Matlab R2017a (MathWorks, Natick, USA) for the brain; the Spinal Cord Toolbox (SCT, version 5.8) for the cSC and the FMRIB Software Library (FSL, version 6.0.5) for image visualisation and signal extraction [11, 12].

#### 2.3.1 Computation of Quantitative Maps

**FIGURE 1:**
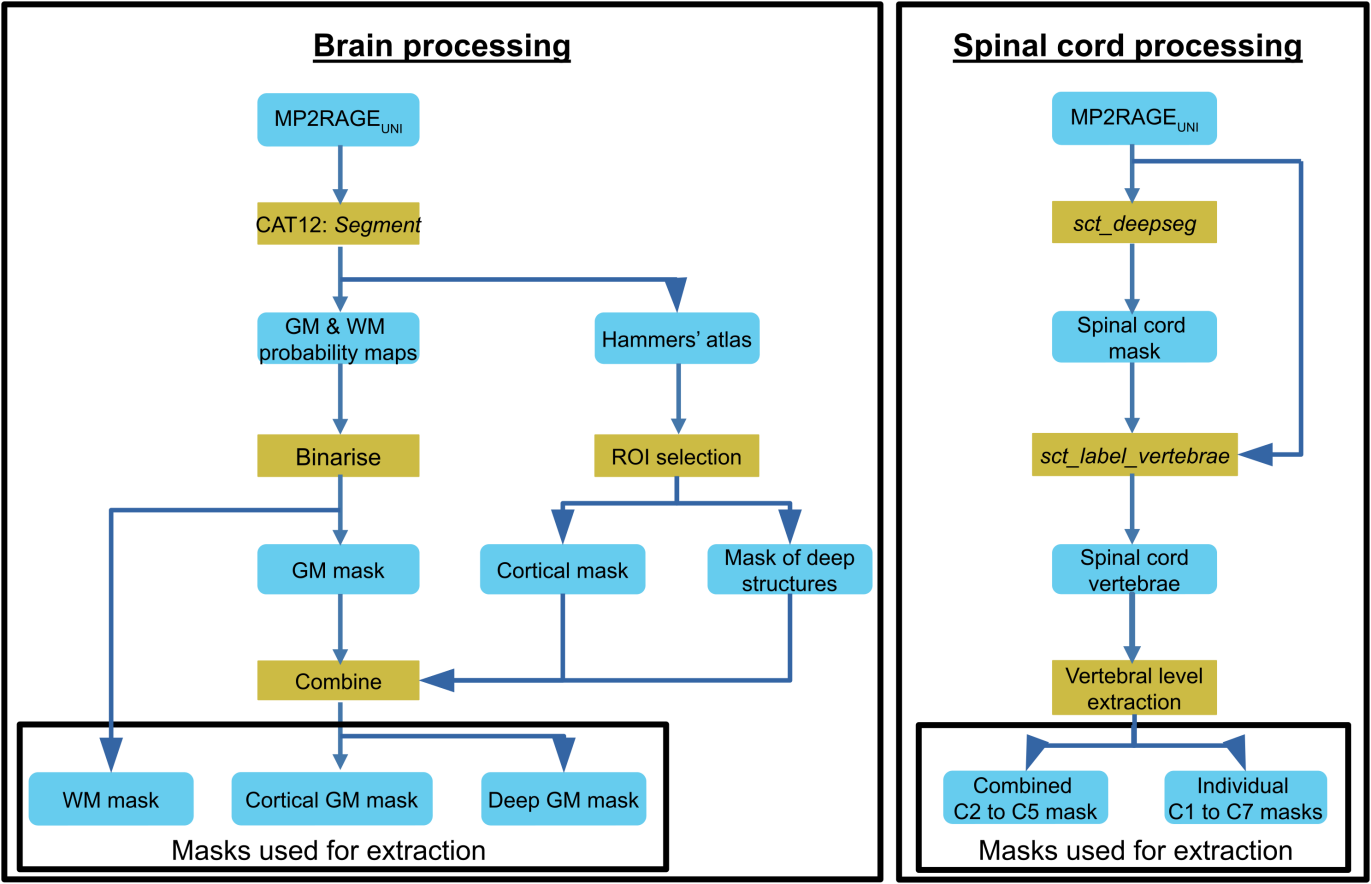
Post‐processing steps for brain and cervical spinal cord, from native MP2RAGE_UNI_ images to masks used for qT1 metrics extraction. Input and output images in the subject space are displayed in blue squares and processing steps are displayed in green squares. Abbreviations: C1 to C7: vertebral levels from cervical C1 to C7; C2 to C5: vertebral levels from cervical C2 to C5; GM = Gray Matter; WM: White Matter.

To reduce the B1 + bias that may exist and would lead to inaccurate T1 estimation if not considered, qT1 maps were generated by integrating the Bloch equations and a look‐up table accounting for B1 + variations [13]. Post‐processing in the brain and the cSC were then performed as described below and in Figure 1.

#### 2.3.2 Brain Post‐Processing

Using the *segment* module of CAT12 (default options but bias correction set at 0.75) based on a refined version of SPM Unified Segmentation on MP2RAGE_UNI_ images [14], probability maps were generated for the three main brain compartments (GM, WM, cerebrospinal fluid). Deformation maps from the Hammers' atlas to MP2RAGE space (based on the Geodesic Shooting approach) were also computed [15, 16]. WM and GM masks were derived from WM and GM probability maps binarized at 0.95 (strict threshold selected to remove any partial volume effect at the transition between compartments) and a cluster size of at least 100 contiguous voxels (to remove rare isolated voxels out of the brain left by CAT12 segmentation). Moreover, two additional brain masks were generated based on Hammer's atlas: a mask of deep structures (including hippocampi, amygdalae, putamens, pallida, thalami, and caudate and accumbens nuclei) and a cortical mask (including frontal, parietal, temporal, insular and occipital cortices). The latter were used in combination with the GM mask to derive two masks for deep GM and for the cortex. Mean qT1 values were then extracted using *fslstats* in three regions of interest (ROI): *WM mask* (including cerebrum, cerebellum and brainstem), *deep GM mask* and *cortical GM mask*.

#### 2.3.3 Cervical Spinal Cord Post‐Processing

Cervical SC were segmented on the MP2RAGE_UNI_ images using the deep learning *sct_deepseg* module with the *seg_ms_sc_mp2rage* model included in the SCT and the default binary mask output [17]. At this stage, some minor corrections in 21/52 segmentations were made when needed, notably filling out missing parts of the axial cSC masks manually at the bottom of C7 (16/21) or at the top of C1 (1/21), or removing extra voxels/slices above C1 (3/21) or below C7 (1/21) to correct the levels. Then, C1 to C7 vertebrae were labelled using the *sct_label_vertebrae* module automatically. If the labelling failed, a semi‐automatic method was used (17/52 scans): by pointing on the intervertebral space between C5 and C6 and rerunning *sct_label_vertebrae*; or if it failed again, by pointing on the intervertebral space between C2 and C3 and rerunning *sct_label_vertebrae*. Ultimately, if the automatic or semi‐automatic methods failed, a manual labelling was performed by pointing at all intervertebral discs between C1 and C7 (19/52 scans). Mean qT1 values were then extracted using *fslstats* in all individual vertebrae from C1 to C7 and in a mask gathering C2 to C5 vertebrae (named C2–C5).

### 2.4 Statistical Analysis

All statistical analyses were performed with R Studio v2024.12.1 (release 563) (www.rstudio.com) software based on R (version 4.5.1; www.r‐project.org). A summary of the subjects used in the analyses was reported in Table S1.

#### 2.4.1 Mean Differences Between Vertebral Levels and Centre Effect

For each scanner, the means and standard deviations (SDs) of qT1 measurements were calculated for the brain ROIs (WM, deep GM and cortical GM) and cSC levels in all subjects scanned (22 scanned in Centre 1 only; nine scanned in Centre 2 only; six scanned at both centres).

Then, to estimate the centre effect in each of the brain ROIs and in the cSC C2–C5 ROIs, linear mixed effect models with participant‐specific random intercepts and centre as predictor, qT1 as dependent variable were fitted (see details in next section) and age as covariate. All subjects seen in one centre only (*n* centre 1/centre 2 = 22/9; the six subjects seen in both centres were not included in the sake of data independence) were included in this analysis. Significance was set to a *p*‐value < 0.05. When applied, Bonferroni corrections for multiple comparisons were reported (*p* < 0.05/10 ROIs = 0.005).

Additionally, a linear mixed effect model was computed with subjects seen in one centre only as a random effect, qT1 as the dependent variable and centre and cSC level as independent variables in order to obtain 95% confidence intervals (CIs) for each vertebra level.

Finally, mean coefficients of variation of each subject were computed in each of the brain ROIs and in the cSC levels for each site for participants seen two times (*n* centre 1/centre 2 = 5/4) to assess intra‐site variability and for the subjects seen in both centres (*n* = 6) to assess inter‐site variability.

#### 2.4.2 Between‐Session and Between‐Participant Variability

**FIGURE 2:**
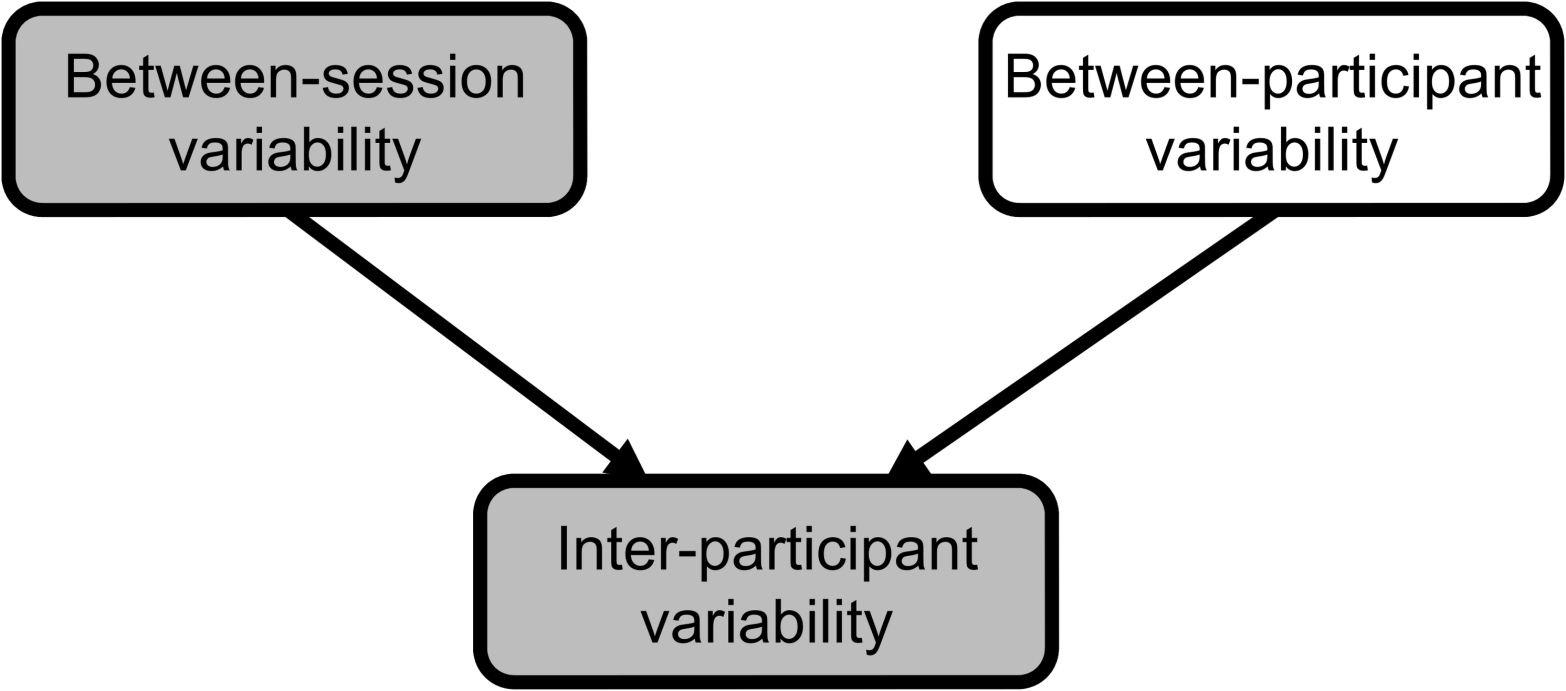
Hierarchical distribution of qT1 variability. The between‐session variability, corresponds to the variability intrinsic to a change of session (i.e., qT1 variability observed when acquiring data on the same subject during two different sessions on the same scanner). The between‐participant variability corresponds to the variability intrinsic to a change of participant (i.e., variability due to the structural and physiological differences between subjects). These two elementary variabilities can be gathered to form inter‐participant variability, characterizing the variability for different subjects in the same scanner. Gray squares represent components directly measurable, whereas the white square represents a component not directly accessible.

Analyses were performed with the same procedure as previously reported [18]. This model is described in Figure 2. As depicted in the figure, the variability of qT1 in a multicentre study can be split into two independent factors: *between‐session variability* and *between‐participant variability, which* can be gathered to form inter‐participant variability, characterizing the variability for different subjects in the same scanner. In contrast to the previous article [18], the *between‐scanner* effect was not computed but added as a fixed effect in the model since the study included only two centres which would lead to underfitting.

Two approaches were used to evaluate these variabilities: first, the direct approach, which is one of the most commonly used methods to assess variability of multicentre quantitative MRI measures and second, the random‐effect approach [19, 20]. The two approaches are briefly described below.

##### 2.4.2.1 Direct Approach

The between‐session SD was estimated from the nine scan‐rescan acquisitions in both centres (*n* Centre 1/Centre 2 *=* 5/4); the inter‐participant SD was estimated from the 43 first scans in both scanners (*n* Centre 1/Centre 2 = 28/15). The associated 95% CIs were computed from the Χ^2^ (chi squared) distribution.

##### 2.4.2.2 Random‐Effect Approach

For each ROI (brain WM, deep GM and cortical GM and cSC C2–C5), a linear random model was used to estimate the maximum likelihood estimation of the variance of the various components, with mean qT1 in the ROI as the dependent variable and participant‐specific random intercept. This second approach used the full dataset including 52 acquisitions to jointly estimate all the components and their 95% CIs.

For both described approaches, results are reported as coefficient of variation (100 × SD/mean) with the associated 95% CI. Moreover, intraclass coefficients (ICC) associated with a given variable were also reported. ICC of a variable represents the proportion of the total variance explained by that variable. Once the above‐defined SDs had been estimated for each of the two approaches, ICCs associated with the between‐session and between‐participant variabilities (and their associated 95% ICs) were calculated as follows:

$$\text{ρSession}=\frac{\delta \text{between}-\text{session}}{\delta \text{overall}}\;$$

$$\text{ρParticipant}=\frac{\delta 2\text{between}-\text{participant}}{\delta 2\text{overall}}$$

with ρ, the ICC, δ2overall, the variance of the overall model, and δ2between−session/δ2between−participant, the variances associated with between‐session and between‐participant variabilities, respectively.

#### 2.4.3 Sample Size Estimates

Considering future applications in a neurodegenerative context, an estimation of the sample sizes needed to reach a Type‐I error of 0.05 and a Type‐II error of 0.2 (one‐tailed) for hypothetical qT1 mean differences between two groups was also computed in all ROIs (brain WM, dGM and cGM, and cSC C2–C5). For example in Multiple Sclerosis, after a search in Pubmed for ‘MP2RAGE and (degenerative or MS)’ (in September 2025), 56 results were returned. Nevertheless, direct comparisons of qT1 between healthy controls and multiple sclerosis patients are scarce and only clearly reported in three published articles for the brain (none for the SC) [21, 22, 23]. The minimal differences described in these articles (37 ms in normal appearing WM [without lesion], 9 ms in dGM [right thalamus only, including lesions, if any] and 8 ms in cGM [including lesions, if any]) [21] were used to estimate the number of subjects required for accurate comparison in the brain. For the cSC, minimal differences in the range of 31 to 67 ms were considered based on values measured in normal appearing GM and WM spinal cord tissues (without lesions) [5]. To account for the variability induced by the pathology, the SD in the patient group was considered as twice bigger than in the control group (about 15 ms in brain WM and cortex, 28 ms in dGM [21] and 23 ms in cSC [5]).

#### 2.4.4 Effect of ROI Size

To test the impact of the brain size as compared to the SC, two small‐size ROIs located in the brain at the interface with CSF were considered, namely the genu and the splenium parts of the corpus callosum (CC) from Johns Hopkins University (JHU) atlas [24]. Moreover, to compare with the spinal cord postprocessing methodology, no threshold was used to binarize the masks. For each scanner, the means and standard deviations (SDs) of qT1 measurements in these regions were reported. Mean coefficients of variation of each subject were computed in each of the CC ROIs for the subjects seen in both centres (*n* = 6) to assess the inter‐site variability.

## 3 Results

**FIGURE 3:**
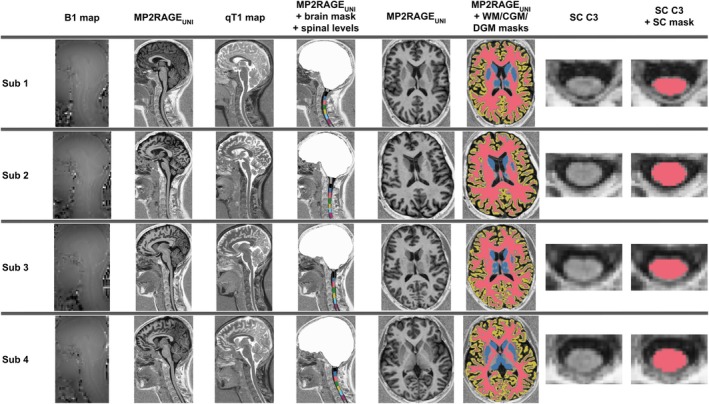
Representative B1 and MP2RAGE maps collected in four different subjects (Sub/Age(y)/Sex: Sub1/30/F, Sub2/50/M, Sub3/32/F and Sub4/24/M). Sagittal B1map, MP2RAGE_UNI_ and qT1 map are represented in Columns 1, 2 and 3, respectively. Sagittal views of brain (white) and cervical levels (coloured) are presented in Column 4. Axial views (UNI image and masks) in the brain and C3 cervical level are presented on the last four columns. Abbreviations: CGM = cortical gray matter, DGM = deep gray matter, qT1 = quantitative T1, SC C3 = Spinal Cord C3 level, Sub = Subject, WM = white matter.

**FIGURE 4:**
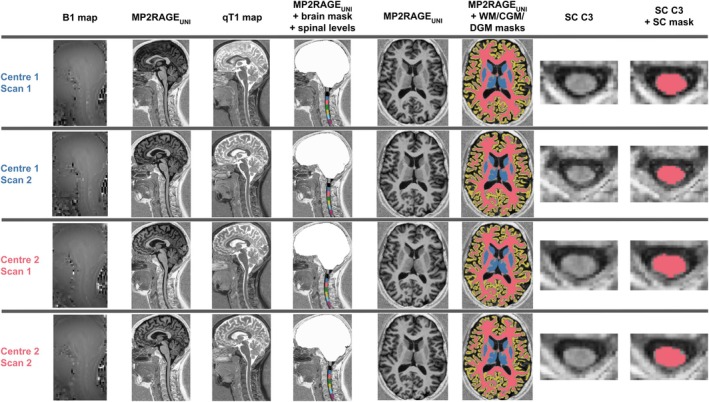
Representative B1 and MP2RAGE maps collected during the scan‐rescan and the inter‐site sessions in one single subject. Lines 1 and 2 depicted the scan‐rescan of Centre 1 (in blue), Lines 3 and 4 depicted the scan‐rescan in Centre 2 (in red). Sagittal B1map, MP2RAGE_UNI_ and qT1 map are represented in Columns 1, 2 and 3, respectively. Sagittal views of brain (white) and cervical levels (coloured) are presented in column 4. Axial views (UNI image and masks) in the brain and C3 cervical level are presented on the last four columns. Abbreviations: CGM = cortical gray matter, DGM = deep gray matter, qT1 = quantitative T1, SC C3 = Spinal Cord C3 level, Sub = Subject, WM = white matter.

Figure 3 illustrates representative B1+, MP2RAGE_UNI_ and qT1 maps obtained from four individual participants, whereas Figure 4 presents the same maps but obtained in one single subject during the scan‐rescan and inter‐site sessions.

All data were carefully quality‐checked. Some of the data were slightly artefacted (e.g., see Figure 3, Subject 2), especially at the lower cervical level due to respiratory motion. Nonetheless the 52 scans were used for the analysis.

### 3.1 Mean qT1 in the Two Centres

**FIGURE 5:**
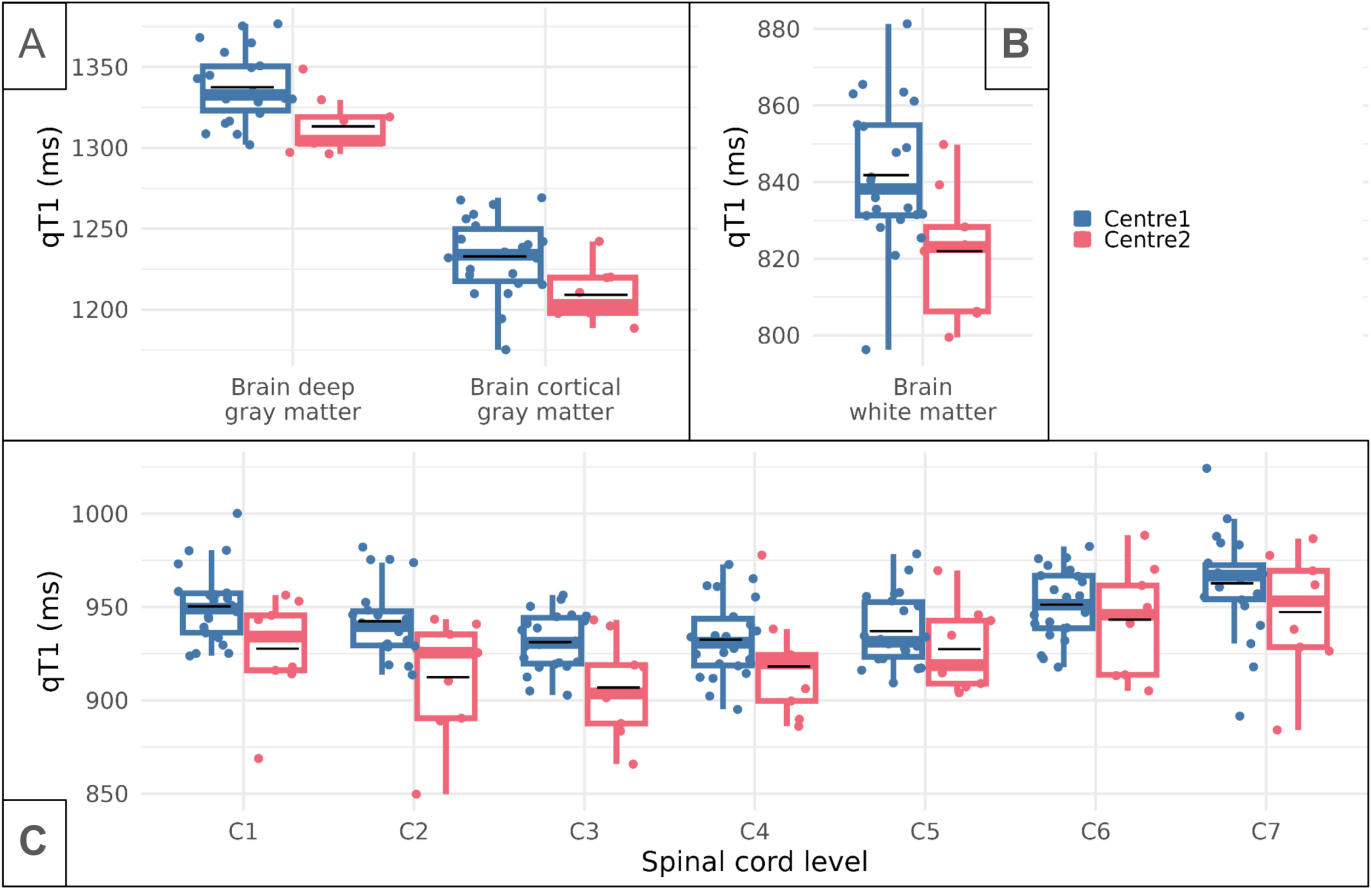
Boxplots of mean qT1 measures in the brain cortical and deep GM (A) and WM (B), and in the cervical spinal cord levels (C) for the acquisition of all subjects seen one time, in Centre 1 only (*n* = 22, blue) or in Centre 2 only (*n* = 9, red). One dot represents one subject. Median, first and last quartiles are represented in the boxplots and means are represented with black lines.

Table 2 and Figure 5 display the mean qT1 measures obtained in each centre, for the selected ROIs, pooling the subjects that were scanned once only. For all regions of the brain, we observed a higher mean value in Centre 1 than in Centre 2 with differences ranging from 20 ms (normalised difference: 2.4%) for WM to 24 ms (normalised difference: 2.0%) for cortical GM. The same observation was made for the spinal cord with mean differences ranging from 8 ms (normalised difference: 0.8%) for C6 to 30 ms (normalised difference: 3.2%) for C2.

**TABLE 2:** Mean qT1 values in each centre for each region of interest (22 subjects with mean age = 42.0 ± 12.6 years [range = [26–62], median = 40] scanned in Centre 1 only and nine subjects with mean age = 34.9 ± 8.8 years [range = [24–48], median = 33] scanned in Centre 2 only; the six subjects scanned at both centres were not considered here) and differences between the two centres based on linear mixed effect model. Mean difference (SD, mean normalised differences) between Centres 1 and 2 (first scan only) for the *n* = 6 subjects scanned at both centres are provided as well in the last column for comparison, confirming that the observed between‐centre differences reflect scanner or site effects and were not affected by the different ages of the cohorts in the different centres. Normalised differences are computed as 2 * (mean qT1 in Centre 1 − mean qT1 in Centre 2) / (mean qT1 in Centre 1 + mean qT1 in Centre 2) * 100. Abbreviations: GM = gray matter, ms = milliseconds, SC = spinal cord, SD = standard deviation, WM = white matter, * *p*‐value surviving the correction for multiple comparisons (Bonferroni correction based on the 10 ROIs tested).

Mean qT1 (SD), in ms	Centre1, *n* = 22	Centre2, *n* = 9	Difference, in ms [normalised difference, in %]	*p* ^a^	Mean difference in ms [SD, mean normalised differences in %] between Centres 1 and 2 (first scan only) *n* = 6
Brain WM	842 (19)	822 (16)	20 [2.4]	< 0.001*	4.4 [2.2, 0.5%]
Deep GM	1233 (24)	1209 (16)	24 [2.0]	< 0.001*	14.5 [6.2, 1.2%]
Cortical GM	1337 (22)	1313 (17)	24 [1.8]	< 0.001*	16.7 [7.7, 1.3%]
Spinal cord C1	950 (20)	928 (27)	23 [2.3]	0.003*	11.9 [26.3, 1.3%]
Spinal cord C2	942 (19)	912 (31)	30 [3.2]	0.026	4.9 [19.2, 0.5%]
Spinal cord C3	931 (16)	907 (26)	24 [2.6]	0.023	−0.5 [26.0, −0.1%]
Spinal cord C4	933 (21)	918 (28)	14 [1.6]	0.058	3.6 [27.1, 0.4%]
Spinal cord C5	937 (19)	927 (22)	10 [1.1]	0.127	6.3 [25.9, 0.7%]
Spinal cord C6	951 (19)	943 (28)	8 [0.8]	0.002*	28.3 [23.9, 3.0%]
Spinal cord C7	963 (27)	947 (32)	15 [1.7]	0.012	14.4 [27.7, 1.5%]
Spinal cord C2–C5	936 (15)	916 (25)	20 [2.2]	0.016	3.8 [18.5, 0.4%]

^**a**^ Computed from the linear mixed effect model with qT1 as dependent variable, participant as random effect, and centre as predictor. Age was also included as covariate.

The analysis from the linear mixed effect models evidenced statistical differences between the two centres in all brain and cSC ROIs (all *p*'s < 0.05), except for the C5 level (*p* = 0.162). Only differences in the brain, and the cSC C1 and C6 ROIs survived the correction for multiple comparisons. For the sake of completeness, a global linear mixed‐effects model with centre and region of interest (WM, deep GM, cortical GM and SC C1 to C7 covariates) as fixed factors and subject as random effect was computed and returned a significant centre effect (*p* = 0.003).

### 3.2 qT1 Variations Along the Cord

**FIGURE 6:**
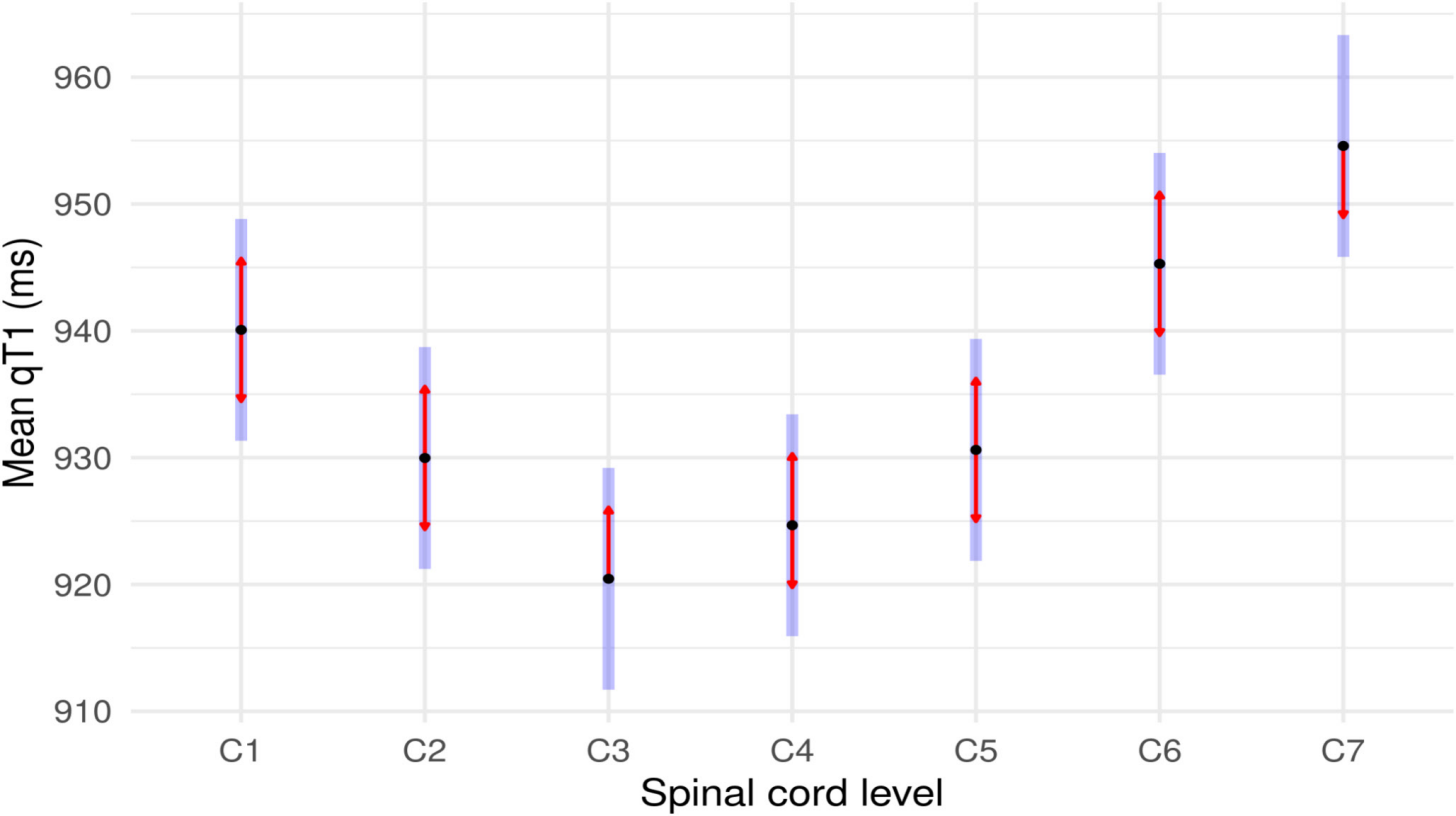
Confidence intervals and differences of mean qT1 (in ms) between spinal cord vertebral levels (pooling both centres and subjects scanned once only [either at Centre 1 or 2; *n* = 31 in total]). Blue bars represent 95% CIs for the mean qT1 measurement. The red arrows indicate significant differences between levels (with Tukey adjustment to control for 5% probability of Type‐I error): if an arrow from one level overlaps with an arrow from another level, the difference is not significant.

No statistical differences were observed between the qT1 means estimated at the C2, C3, C4 and C5 cSC levels. Nonetheless, significant differences were found between qT1 means in these levels and C6, and between these levels and C7 (Figure 6).

### 3.3 Intra‐ and Inter‐Site Repeatability (COV% Evaluation)

Mean intra‐site coefficients of variation were all below 0.34% for the brain ROIs in both centres, whereas for cSC these values ranged from 0.98 (C1) to 1.95% (C4) in Centre 1 and from 0.63 (C3) to 1.47% (C5) in Centre 2 (Table 3). Mean inter‐site coefficients of variation were below 0.90% for brain ROIs and ranged from 1.18 (C2) to 2.37% (C6) in the spinal cord. Mean qT1 values and B1 + inhomogeneities measured in the subjects that had the scan‐rescan inter‐site explorations are provided in the Supporting Information (Figures S2–S6).

**TABLE 3:** Mean coefficients of variation (in %) of qT1 values intra‐ and inter‐site for each region of interest. Abbreviations: GM = gray matter, max = maximum, min = minimum, SC = spinal cord, SD = standard deviation, WM = white matter. Subjects were scanned twice intra‐site.

Mean Coefficients of variation (SD) [min–max], in %	Intra‐site—Centre1, *n* = 5	Intra‐site—Centre2, *n* = 4	Inter‐site, *n* = 6
Brain WM	0.22 (0.17) [0.01–0.43]	0.10 (0.12) [0.01–0.27]	0.38 (0.18) [0.09–0.61]
Deep GM	0.14 (0.05) [0.05–0.18]	0.22 (0.13) [0.06–0.37]	0.85 (0.35) [0.38–1.46]
Cortical GM	0.29 (0.36) [0.02–0.90]	0.33 (0.18) [0.13–0.49]	0.89 (0.41) [0.24–1.40]
Spinal cord C1	0.88 (0.76) [0.06–1.83]	0.83 (0.35) [0.60–1.35]	1.82 (1.00) [0.57–3.40]
Spinal cord C2	0.91 (0.74) [0.16–1.83]	0.90 (0.57) [0.05–1.24]	1.18 (0.77) [0.14–2.06]
Spinal cord C3	1.22 (1.01) [0.06–2.63]	0.63 (0.44) [0.30–1.26]	1.47 (1.17) [0.02–3.49]
Spinal cord C4	1.72 (1.52) [0.13–4.14]	0.85 (0.75) [0.24–1.87]	1.79 (0.83) [0.84–2.71]
Spinal cord C5	1.07 (1.02) [0.02–2.49]	1.47 (1.07) [0.47–2.64]	1.42 (1.43) [0.07–4.16]
Spinal cord C6	0.90 (0.77) [0.15–2.00]	1.33 (0.77) [0.35–2.18]	2.37 (1.44) [0.44–3.82]
Spinal cord C7	1.37 (1.04) [0.27–3.09]	1.05 (0.72) [0.07–1.72]	1.95 (0.98) [0.94–3.42]
Spinal cord C2–C5	1.13 (1.13) [0.01–2.90]	0.75 (0.90) [0.06–2.05]	1.14 (0.77) [0.00–2.07]

### 3.4 Between‐Session and Between‐Participant qT1 Variability

**FIGURE 7:**
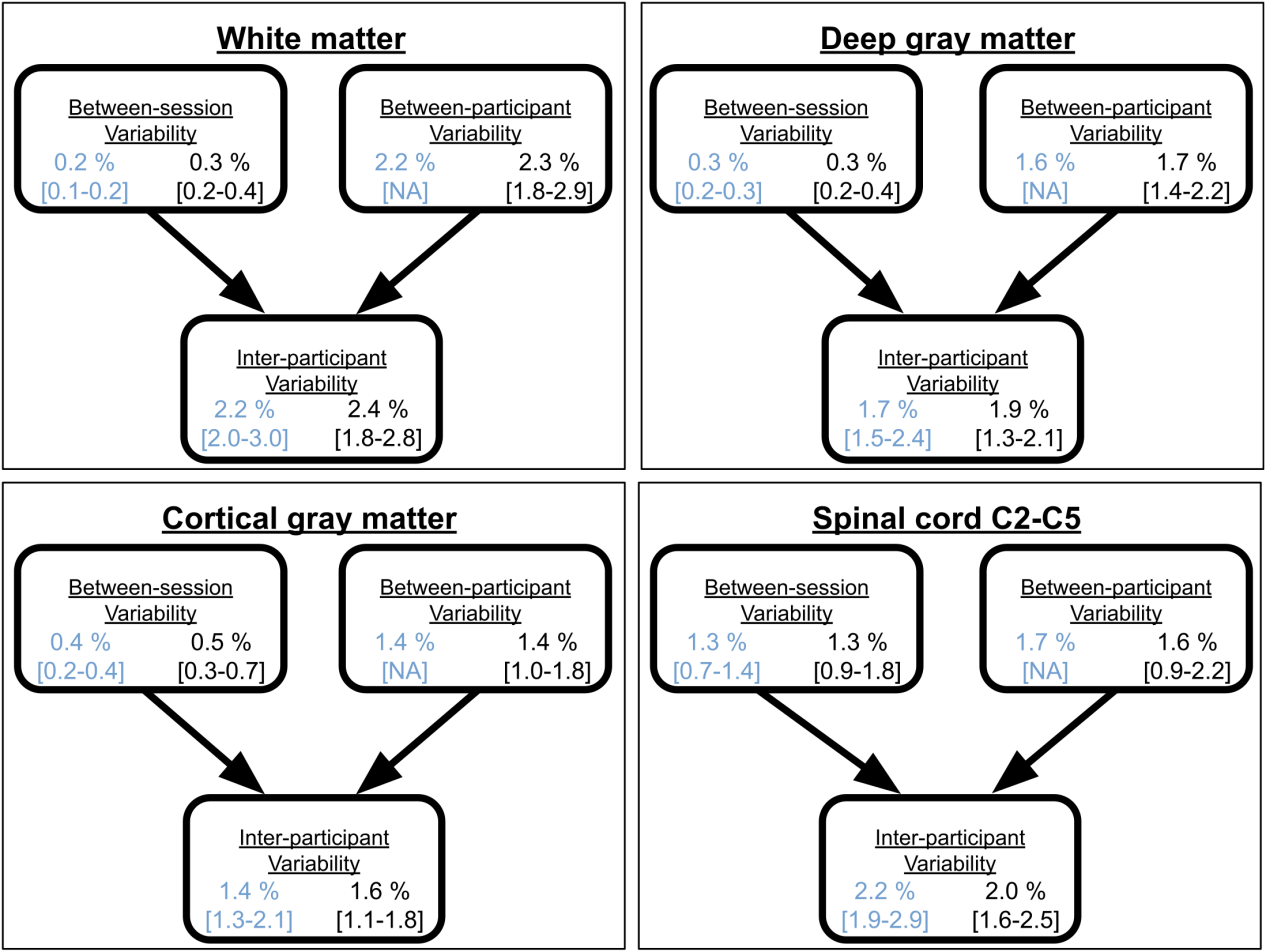
Coefficients of variation and associated 95% CIs of mean qT1 for the variability estimated using either the direct (in light blue) or random‐effect (in black) approach.

All results for the coefficient of variation (CoV) are summarised in Figure 7 for direct and random‐effect approaches. Both approaches displayed similar results.

For the brain WM, deep and cortical GM, the between‐session CoVs were 0.2%, 0.3% and 0.4%, respectively. The between‐participant CoVs were 2.2%, 1.6% and 1.4%, respectively. For the C2–C5 cSC ROI, the between‐session CoV was 1.3% and the between‐participant CoV was 1.7%.

The corresponding ICCs are displayed in Table 4. Similar values were observed for both direct and random‐effect approaches. For the brain in WM, deep and cortical GM, the between‐session ICCs were 0.01, 0.02 and 0.08 with the direct approach, and the between‐participant ICCs were 0.99, 0.98 and 0.92, respectively. For the cSC C2–C5 ROI, the between‐session ICC was 0.35 and the between‐participant CoV was 0.65. Thus, the total variance in these ROI was mainly explained by between‐participant variation, especially for the brain ROIs.

**TABLE 4:** Intraclass coefficients (ICCs) and associated 95% confidence intervals (only possible with the random‐effect approach) of mean qT1 for the between‐session and between‐participant components.

	Between‐session intraclass coefficient	Between‐participant intraclass coefficient
White matter		
Direct approach	0.01 [NA]	0.99 [NA]
Random‐effect approach	0.01 [0.004, 0.027]	0.99 [0.97, 1.0]
Deep gray matter		
Direct approach	0.02 [NA]	0.98 [NA]
Random‐effect approach	0.02 [0.009, 0.06]	0.98 [0.94, 0.99]
Cortical gray matter		
Direct approach	0.08 [NA]	0.92 [NA]
Random‐effect approach	0.11 [0.04, 0.23]	0.89 [0.76, 0.96]
Spinal cord C2–C5		
Direct approach	0.35 [NA]	0.65 [NA]
Random‐effect approach	0.39 [0.15, 0.86]	0.61 [0.12, 0.85]

### 3.5 Sample Size Estimation

**FIGURE 8:**
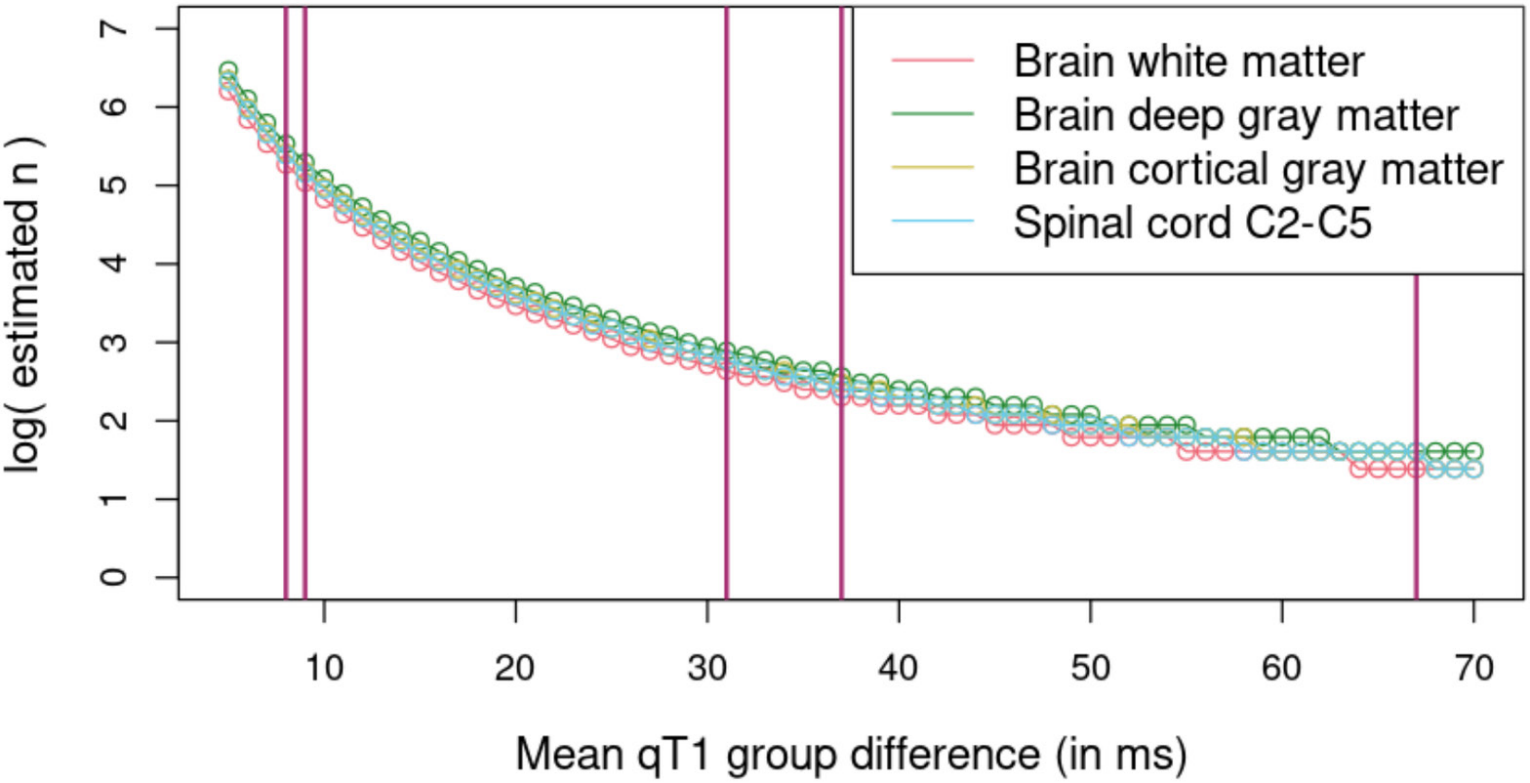
Estimated n (expressed as log transformed n) associated with expected mean qT1 group difference for brain white matter (red line and dots), deep gray matter (green line and dots), cortical gray matter (yellow line and dots) and C2–C5 spinal cord (blue line and dots). Purple vertical lines depict the expected mean qT1 differences for cortical gray matter (8 ms corresponding to an *n* = 225), deep gray matter (9 ms corresponding to an *n* = 199), brain WM (37 ms corresponding to an *n* = 10) and spinal cord C2–C5 (31 or 67 ms corresponding to an *n* = 16 or 5).

Sample size estimations to detect group differences ranging from 5 to 70 ms are reported in Figure 8. The estimated number of subjects per group was 10, 199, and 225 to detect differences of 37, 9 and 8 ms in brain WM, dGM and cGM, respectively. For C2–C5 cSC, five or 16 subjects per group would be necessary to observe a statistical difference of 31 to 67 ms between groups, respectively.

### 3.6 Effect of ROI Size

The genu and splenium CC had approximately the same size as the C2–C5 cervical SC (average volume of ~7.000 mm^3^ for the genu and 9.000 mm^3^ for the splenium vs. 4.100 mm^3^ for the C2–C5 cervical SC). The mean qT1 values were higher in the Centre 1 compared to Centre 2 for both regions [mean qT1 in Centre 1/2: 992.2 (SD: 207.8)/981.6 (SD: 218.5) ms in the genu of the CC and 882.2 (SD: 104.7)/868.7 (SD: 95.8) ms in the splenium of the CC]. COV between the two centres was 1.03 (SD = 0.48) and 1.04 (SD = 0.59) for the genu and the splenium of the CC, respectively.

## 4 Discussion

This study evaluates the repeatability and reproducibility of simultaneous brain and cervical spinal cord MP2RAGE T1 values measured in healthy controls in two different MR centres.

### 4.1 Mean qT1 Values and Inter‐Session COV

Mean qT1 values of 842/822, 1337/1313, 1233/1209, and 936/916 ms in Centre 1/Centre 2 were obtained in the brain WM, cortical GM, deep GM, and C2–C5 spinal cord, respectively. The qT1 values along the spinal cord exhibited a U‐shape distribution, with lower values at the C3–C4 level, previously described and linked to the dense WM tracts at the location of the brachial plexus [13].

Measured mean intra‐site inter‐session variabilities were below 0.4% and 1.5% on average in the brain and spinal cord, respectively, and maximal values of 0.9% and 4.1%, respectively, were observed at the individual scale.

The mean T1 and COV values found it this study are in agreement with previous results observed in single‐site studies with MP2RAGE, such as Rasoanandrianina et al. [2], who reported mean qT1 of 943/919 ms in the GM/WM spinal cord and COV of 2.5%/1.9%, or Marques et al. [1] who reported values of 810 and 1350 ms in the brain WM and GM. Agreement with T1 values reported in the literature is not discussed further here, as T1 measurements are inherently dependent on the imaging methodology and differences in imaging protocols can lead to variability in reported T1 values (e.g., due to magnetization transfer effects) [25]. Ultimately, when conducting a multicentre study, the key is to use a standardized or identical protocol, and to assess the repeatability between the centres. So far, and to the best of our knowledge, no such a multicentre study considering both brain and spinal cord was conducted with the MP2RAGE method.

### 4.2 Inter‐Site Repeatability, Inter‐Subject Variations and Total Variance

In this study, using a strictly identical protocol in both centres, mean inter‐site variabilities were observed below 0.9% and 2.4% in the brain and spinal cord, respectively, with maximal values of 1.5% and 4.2% at the individual scale.

Interestingly, in the inter‐site scan‐rescan study, higher T1 values were majoritarily observed in Centre 1 as compared to Centre 2 (cf. Supporting Information, with a bias of 4.44, 16.71, 14.55 and 3.76 for brain WM, cGM and dGM, and SC C2–C5, respectively). Identical product sequences (MP2RAGE and B1 + mapping) and sequence parameters (including RF pulse mode), as available from the software interface, were applied, yet two different scanner models and software versions (Prisma/VE11 and Vida/XA50) were used, with possible minor changes in the sequence implementations, which we cannot control nor access. Additional testing using a well‐controlled and heatable T1 phantom and experiments in centres equipped with the same software versions would be ideal to fully investigate the differences.

Besides, when comparing the different cohorts (Table 2), mean qT1 differences between centres were observed, some of which were statistically significantly different, yet all with a constant and mild difference of around 2%. As previously reported [3, 26], qT1 values vary with age and the slight difference in age mean and ranges could also explain some of the difference observed between the two centres.

Altogether, the total variance in the qT1 measurements was mainly explained by the inter‐participant variability, with an ICC of 0.99 in the brain, and 0.65 in the spinal cord. Challenges related to spinal cord imaging and data analysis, subject repositioning, shape of the organ, small cross‐sectional area and physiological motion all contribute to the between‐session variability, including partial volume effect or segmentation and registration bias.

In the same way, we checked for the impact of ROI size in brain vs. spinal cord by considering two small brain regions, namely the genu and the splenium of the CC. The intra‐centre COVs (1.03 [SD = 0.48] and 1.04 [SD = 0.59], for the genu and the splenium of the CC) were higher in both ROIs than what was observed in the brain WM (0.38 [SD = 0.18]) and slightly lower than what was observed in the C2–C5 spinal cord (1.14 [SD = 0.77]). These results highlighted that the size of the ROI may not have a direct impact on the qT1 signal, but that it is more a combination of different factors such as segmentation accuracy, mask threshold, partial volume effect, and slight physiological variations.

Nonetheless, results obtained in this study are very promising for future multicentric studies focusing on traumatic or neurodegenerative pathologies and MS in particular. Indeed, exploratory studies in the spinal cord, as well as more comprehensive studies in the brain demonstrated pathological changes in normal appearing tissues in the order of 20 ms (cerebral WM) [27] to 60 ms (cSC) [7] in average and much more in the MS lesions (250 ms in SC) [5] up to 380 ms in the brain [27] thus far exceeding the variability measurements observed here.

Altogether, the M2PRAGE sequence thus provides a combined quantification of brain and spinal cord T1 values in a single sequence, allowing both improved lesion detection [4] and assessing tissue microstructure. Having all this information combined in a single 3D sequence is time efficient and optimizes image manipulation. This sequence is an ideal candidate for longitudinal clinical research protocols where biophysical metrics can help follow disease evolution and detect subtle changes.

Nonetheless, this study comes with several limitations. Only two centres with the same manufacturer took part in data collection. Of note, at the time of the study, the MP2RAGE sequence was only available on systems for a single manufacturer, but the sequence is now more widely available. Besides, since qT1 measurements are not absolute measurements, qT1 values may vary with sequence implementation and parameters, making it challenging to pool data acquired on scanners from different manufacturers. Importantly, the same magnetization preparation should be achieved to minimize biases and future studies evaluate the reproducibility between vendors. In the meantime, patient follow‐up should preferentially be restricted to the same scanner or to the same manufacturer. Also, no attempt to separate WM and GM in the spinal cord, nor WM tracts, was performed in this study since this is very dependent on atlas registration and partial volume effects are difficult to avoid. Instead, we therefore chose to compare mean values for each vertebral level only. Moreover, postprocessing (spinal cord segmentation, vertebrae labelling) required manual edits in many cases. New tools such as ‘TotalSpineSeg’ (included in SCT since version 6.5) may be more reliable to achieve robust fully automatic processing. Lastly, in the perspective of the application of the MP2RAGE sequence in the pathological context and more particularly in MS, reproducibility should also be evaluated in patients presenting lesions, to take into account additional variability due to imperfect lesion segmentation and registration issues and answer the ultimate question: how robust and how sensitive is quantitative MRI, in its global process, to follow lesion evolution?

Finally, as a future prospect yet beyond the scope of this work, the interest of using physics‐constrained deep neural networks for multisite and multiscanner MRI datasets harmonization could be investigated as well [28].

In conclusion, the present results confirm the consistency and reliability of the MP2RAGE protocol in a multicentre context provided that the exact same acquisition parameters are implemented. They highlight its potential for further applications in multicentre studies to detect brain and spinal cord lesions, assess macrostructural tissue impairment and provide advanced diagnosis and prognosis tools for CNS pathologies.

## Funding

This research was supported by the Foundation France Multiple Sclerosis (France SEP), the Institute for Clinical Neuroscience of Rennes (INCR), the CORECT grant from Rennes University Hospital, and France Life Imaging (FLI) (ANR‐11‐INBS‐0006).

## Conflicts of Interest

The authors declare no conflicts of interest.

## Supporting information




**Table S1:** Summary of the data used for each analysis.
**Figure S2:** Bland–Altman plots in brain white matter.
**Figure S3:** Bland–Altman plots in cortical gray matter.
**Figure S4:** Bland–Altman plots in deep gray matter.
**Figure S5:** Bland–Altman plots in spinal cord C2–C5.
**Figure S6:** B1 + inhomogeneities for the subjects scanned in the two centres.

## Data Availability

The data that support the findings of this study are available from the corresponding author upon reasonable request. Supporting Information is available, consisting of a summary table of the data used for each analysis, Bland–Altman plots, and the B1 + inhomogeneity differences between the two centres.
