# Supplementary material for: Multicenter Assessment of Combined Brain and Cervical Spinal cord 3T MP2RAGE T1 Measurements for Reliable Tissue Microstructure Quantification
Source: NMR Biomed. 2026 Jul 8;39(8):e70350. doi: 10.1002/nbm.70350 (PMC13344110; doi:10.1002/nbm.70350)
Supplement: Supplementary file 1 — Table S1: Summary of the data used for each analysis. Figure S2: Bland–Altman plots in brain white matter. Figure S3: Bland–Altman plots in cortical gray matter. Figure S4: Bland–Altman plots in deep gray matter. Figure S5: Bland–Altman plots in spinal cord C2–C5. Figure S6: B1 + inhomogeneities for the subjects scanned in the two centres. [file NBM-39-e70350-s001.docx]

Multicenter assessment of combined brain and cervical spinal cord 3T MP2RAGE T1 measurements for reliable tissue microstructure quantification

Gaubert Malo^1,2^, Combès Benoit^2^, Henitsoa Rasoanandrianina^3,4^, Ferré Jean-Christophe^1,2^, Dufey Alice^5^, Chouteau Raphaël^5^, Bertrand Audoin^3,4,6^, Jean Pelletier^3,4,6^, Sarah Demortière^3,4,6^, Kerbrat Anne^2,5^, Bannier Elise^1,2^, Callot Virginie^3,4^

1 Univ Rennes, CHU Rennes, (Service de radiologie et imagerie médicale), F-35000 Rennes, France

2 University Rennes, Inria, CNRS, Inserm, IRISA UMR 6074, Empenn U1228, Rennes, France

3 Aix-Marseille Univ, CNRS, CRMBM, Marseille, France

4 APHM, Hôpital Universitaire Timone, CEMEREM, Marseille, France

5 Univ Rennes, CHU Rennes, (Service de neurologie), F-35000 Rennes, France

6 APHM, Hôpital Universitaire Timone, Department of Neurology, Marseille, France

Corresponding authors: [elise.bannier@irisa.fr](mailto:elise.bannier@irisa.fr) - [virginie.callot@univ-amu.fr](mailto:virginie.callot@univ-amu.fr)

[Supplementary Table S1: Summary of the data used for each analysis 3](#_irm9zvw12b4z)

[Supplementary Figure S2: Bland-Altman plots in brain white matter 4](#_l6uyj8k8aj9g)

[Supplementary Figure S3: Bland-Altman plots in cortical gray matter 5](#_sw9q06av6lmo)

[Supplementary Figure S4: Bland-Altman plots in deep gray matter 6](#_whi96a8nx4bk)

[Supplementary Figure S5: Bland-Altman plots in spinal cord C2-C5 7](#_7thcgvwj9hkx)

[Supplementary Figure S6: B1+ inhomogeneities for the subjects scanned in the 2 centers 8](#_7081xjkn3tl6)

## Supplementary Table S1: Summary of the data used for each analysis

| **Analyses** | **Subjects** | | | **unit of analysis** | **How repeated scans were used?** |
| --- | --- | --- | --- | --- | --- |
|  | **Scanned once in 1 centre only**  **(n centre 1 / 2=22/9)** | **Scanned 2 times in 1 centre only**  **(n=1)** | **Scanned in both centres, once or 2 times (n=6)** |  |  |
| linear mixed effect models (centre effect) | yes | yes (first scan only) | no | scan | NA |
| a linear mixed effect model (cSC levels) | yes | yes (first scan only) | no | scan | NA |
| COV for intra-site variability | no | yes | yes | scan | average of individual COV for both scans in each center |
| COV for inter-site variability | no | no | yes | scan | average of individual COV for first scans in both centres |
| Global model (Direct approach) | yes | yes | yes | scan | both scans: between session variability  first scan only: between participant and inter-participant variabilities |
| Global model (random effect approach) | yes | yes | yes | scan | participants as random intercept |

*Summary of the subject participants and how they were used in the analyses. COV : coefficient of variation; NA : not applicable.*

## Supplementary Figure S2: Bland-Altman plots in brain white matter


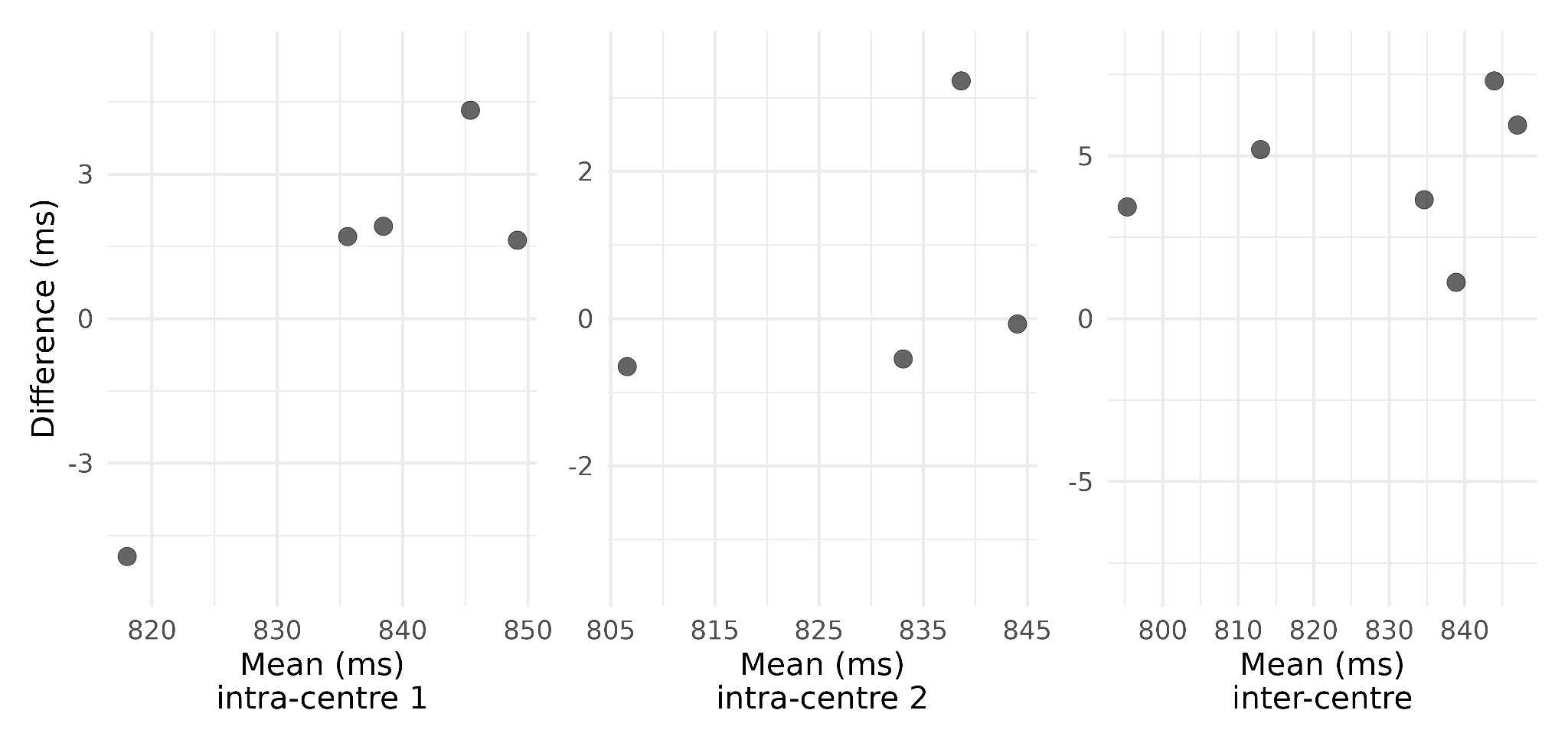


*Bland-Altman plots for qT1 values in brain white matter, comparing intra-centre values in centre 1 (left), centre 2 (centre) and inter-centre values (right) . Bias and limits of agreement [-2 SD - 2 SD] are 0.93 [-6 - 7.86], 0.49 [-3.2 - 4.18] and 4.44 [0.09 - 8.8] for intra-centre in centre 1 and 2, and inter-centre, respectively. One dot represents one subject.*

##

## Supplementary Figure S3: Bland-Altman plots in cortical gray matter


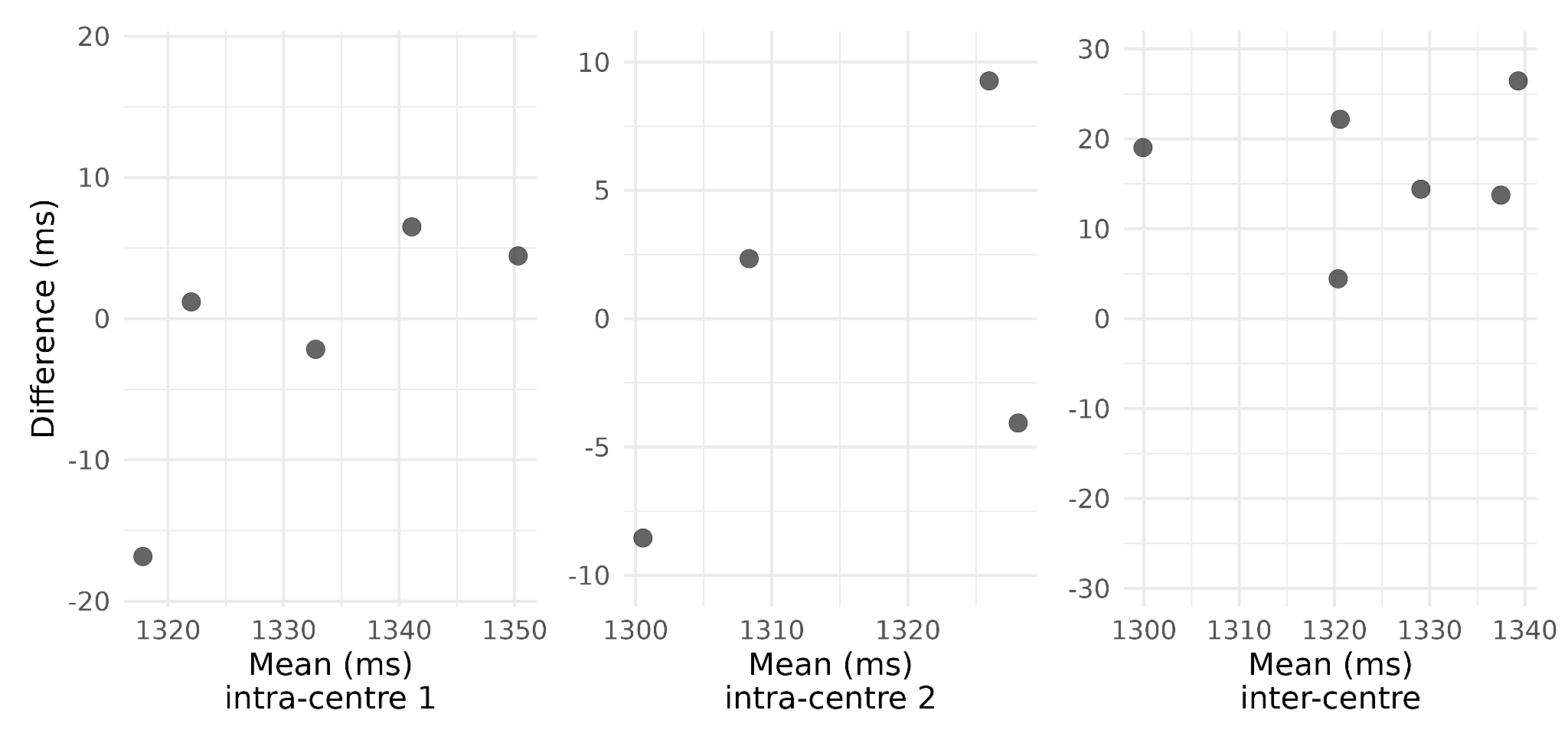


*Bland-Altman plots for qT1 values in cortical gray matter, comparing intra-centre values in centre 1 (left), centre 2 (centre) and inter-centre values (right). Bias and limits of agreement [-2 SD - 2 SD] are -1.37 [-19.87 - 17.13], -0.25 [-15.77 - 15.27] and 16.71 [1.36 - 32.06] for intra-centre in centre 1 and 2, and inter-centre, respectively. One dot represents one subject.*

## Supplementary Figure S4: Bland-Altman plots in deep gray matter


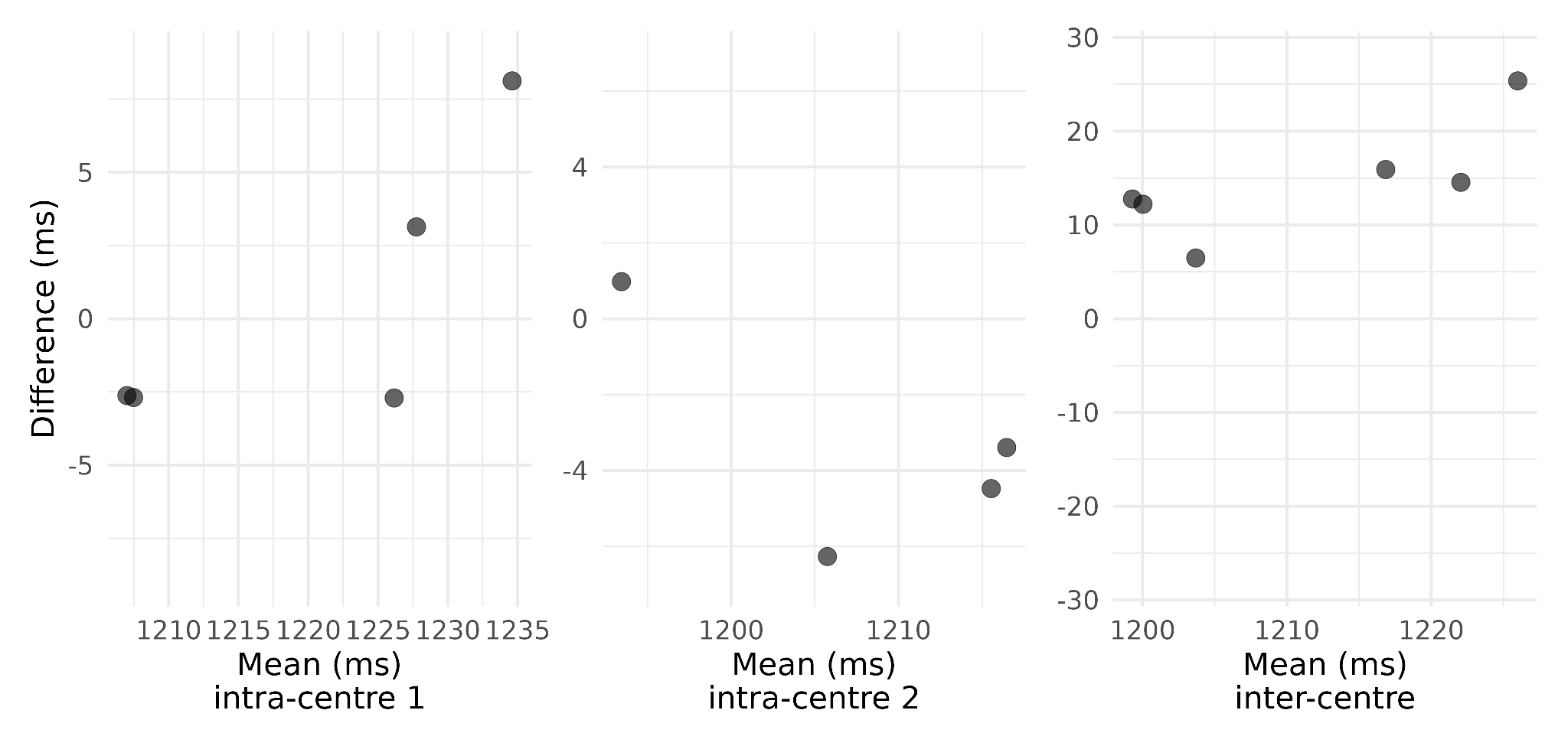


*Bland-Altman plots for qT1 values in deep gray matter, comparing intra-centre values in centre 1 (left), centre 2 (centre) and inter-centre values (right). Bias and limits of agreement [2 SD - 2 SD]) are 0.65 [-9.11 - 10.4], -3.29 [-9.46 - 2.88] and 14.55 [2.31 - 26.96] for intra-centre in centre 1 and 2, and inter-centre, respectively. One dot represents one subject.*

## Supplementary Figure S5: Bland-Altman plots in spinal cord C2-C5


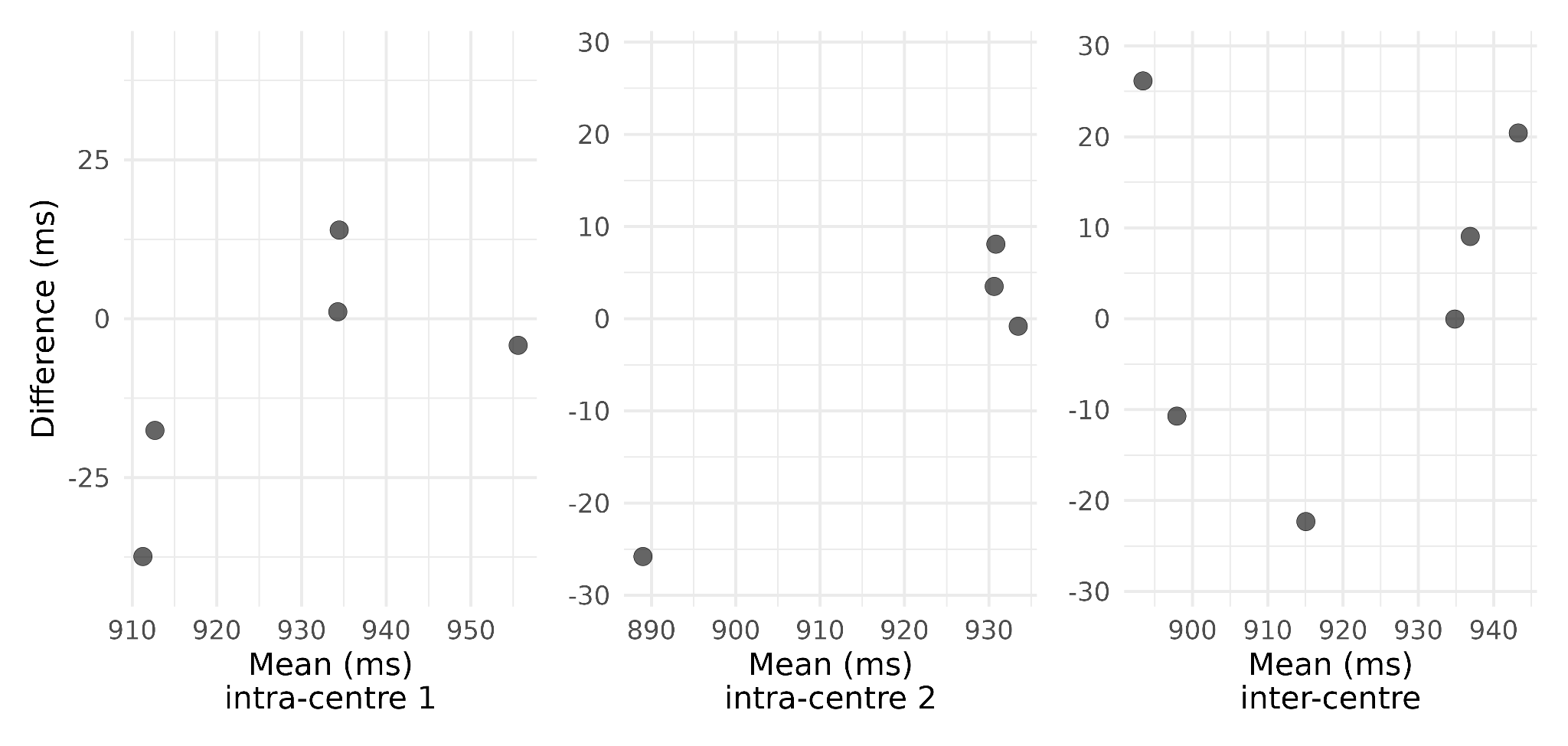


*Bland-Altman plots for qT1 values in spinal cord C2-C5, comparing intra-centre values in centre 1 (left), centre 2 (centre) and inter-centre values (right). Bias and limits of agreement [2 SD - 2 SD]) are -8.82 [-47.96 - 30.32], -3.75 [-34.03 - 26.52] and 3.76 [-33.22 - 40.75] for intra-centre in centre 1 and 2, and inter-centre, respectively. One dot represents one subject.*

## Supplementary Figure S6: B1+ inhomogeneities for the subjects scanned in the 2 centers

| Subject ID | B1+ inhomogeneities (%) in center 1 at C1/C3/C7 | B1+ inhomogeneities (%) in center 2 at C1/C3/C7 |
| --- | --- | --- |
| 1 | 20.3 / 11.5 / -3.3 | 16.1 / 8.0 / -14.2 |
| 2 | 21.8 / 11.8 / -7.8 | 12.8 / 2.9 / -18.8 |
| 3 | 17.5 / 8.3 / -13.1 | 14.0 / 4.4 / -19.4 |
| 4 | 15.8 / 7.3 / -13.0 | 13.1 / 4.7 / -14.7 |
| 5 | 27.9 / 15.8 / -3.5 | 13.4 / 1.6 / -26.2 |
| 6 | 21.2 / 14.7 / -5.8 | 11.3 / 3.2 / -19.7 |

*B1+ inhomogeneities (expressed in %, as compared to the reference target value (80°)) measured at C1, C3 and C7, for the 6 subjects scanned in the 2 centers. B1+ inhomogeneities between the 2 centers slightly differ (with a shift toward positive values and smaller B1+ heterogeneities across the cord (C1-C7) for center 1), likely reflecting differences in the transmit coil efficiency and automatic adjustment strategy (eg. ROI considered for the automatic adjustment). Nonetheless, these differences do not impact T1 values since B1+ heterogeneities are accounted for when calculating T1s.*
